# COVID-19 pandemic’s impact on networks of depression and anxiety in naturalistic transdiagnostic sample of outpatients with non-psychotic mental illness

**DOI:** 10.3389/fpsyt.2023.1118942

**Published:** 2023-03-13

**Authors:** Shin Tae Kim, Jun Ho Seo, Chun Il Park, Se Joo Kim, Jee In Kang

**Affiliations:** ^1^Department of Psychiatry, Yonsei University College of Medicine, Seoul, Republic of Korea; ^2^Institute of Behavioral Science in Medicine, Yonsei University College of Medicine, Seoul, Republic of Korea; ^3^Department of Psychiatry, Yonsei University Wonju College of Medicine, Wonju, Kangwon, Republic of Korea; ^4^Department of Psychiatry, CHA Bundang Medical Center, CHA University, Seongnam, Republic of Korea

**Keywords:** depression, anxiety, COVID-19, pandemic, network analysis

## Abstract

**Background:**

The 2019 coronavirus disease (COVID-19) pandemic has caused an unprecedented disruption of daily lives and a mental health crisis. The present study examined how the depression and anxiety symptom network changed during the COVID-19 pandemic in a naturalistic transdiagnostic sample with non-psychotic mental illness.

**Materials and methods:**

A total of 224 psychiatric outpatients before the pandemic and 167 outpatients during the pandemic were included in the study and were assessed for the Patient Health Questionnaire and the Beck Anxiety Inventory. The network of depression and anxiety symptoms before and during the pandemic were estimated separately and were assessed differences.

**Results:**

The network comparison analysis showed a significant structural difference between the networks before and during the pandemic. Before the pandemic, the most central symptom in the network was feelings of worthlessness, while in the during pandemic network, somatic anxiety emerged as the most central node. Somatic anxiety, which showed the highest strength centrality during the pandemic, showed significantly increased correlation with suicidal ideation during the pandemic.

**Limitations:**

The two cross-sectional network analyses of individuals at one point in time cannot demonstrate causal relationships among measured variables and cannot be assumed to generalize to the intraindividual level.

**Conclusion:**

The findings indicate that the pandemic has brought a significant change in the depression and anxiety network and somatic anxiety may serve as a target for psychiatric intervention in the era of the pandemic.

## 1. Introduction

The 2019 coronavirus disease (COVID-19) outbreak has caused an unprecedented disruption of daily lives and a mental health crisis. As COVID-19 has become a global pandemic with rapidly increasing infection and death rates, mental health problems including fear of being infected and COVID-19-related anxiety have increased (1). In addition, environmental factors that may influence mental health, such as stay-at-home measures and social distancing, have drastically changed. It is crucial to understand the impact of pandemic on mental health outcomes, which may aid in providing effective intervention strategies in the context of the pandemic.

Although research systematically comparing the characteristics of psychiatric disorders before and during the COVID-19 pandemic is sparse, numerous studies on the impact of the pandemic on global mental health have been reported. The 2020 global burden of disease study showed that there was a 27.6% increase in the cases of major depressive disorder (MDD), and an 25.6% increase in anxiety disorders globally due to the COVID-19 pandemic (2). Meta-analyses of general populations have indicated that when comparing psychiatric symptoms before and during the first year of the pandemic, the largest increase was in depression or anxiety symptoms, while psychotic symptoms seemed to decrease slightly (3). A meta-analysis of patients with pre-existing mental and physical conditions during the early pandemic showed that a quarter of them reported anxiety, depression, and stress symptoms, while nearly three quarters experienced sleep problems (4). In addition, insomnia and somatic symptoms were commonly observed in general populations (5), as well as among health professionals (6), during the ongoing COVID-19 pandemic in which anxiety, insomnia, and somatic symptoms have been shown to be closely intercorrelated (7).

Notably, depression and anxiety disorders often co-occur and have considerable symptom overlap, despite separate diagnostic categories (8). Indeed, in clinical practice, patients rarely present with “pure” forms of the disorders and often report a combination of depressive, anxious, and somatic symptoms (9). In addition, depression and anxiety have a complex and multidimensional construct. Defining depression as a disorder is based on symptoms forming a syndrome and limiting psychosocial functioning; some cognitive-affective symptoms are more specific to MDD, such as sad mood, anhedonia, and guilt, while somatic symptoms of depression, such as fatigue, loss of appetite, and insomnia, are common in other medical illnesses as well as other psychiatric disorders, including anxiety and somatic symptom disorders (10, 11). Anxiety encompasses cognitive and somatic components (12); cognitive anxiety reflects thought process-related symptoms, including worry, intrusive thoughts, and fear of losing control, while somatic anxiety reflects muscle tension and physiological arousal, including palpitations and trembling. Since these symptoms of depression and anxiety have been shown to be influenced by the environment (13), they would likely be affected by the COVID-19 pandemic, a period of threat to and uncertainty surrounding health. In particular, during the ongoing pandemic, with increases in the prevalence of physical diseases and in the number of deaths worldwide, individuals’ worry about their physical health might rise, which may lead to an increase in somatic anxiety. Understanding of the patterns of changes in the depression-anxiety relationship over the course of the COVID-19 pandemic could be helpful to mental health professionals and governments in developing mental health services and policies.

For this purpose, network analysis may be useful, which is a new approach to examine the dynamic relationships among different psychopathologies (14). In contrast to the traditional approach to mental illnesses that involve summing up symptoms to establish diagnoses, in network analysis, psychiatric disorders are assumed to stem from a causal interplay between symptoms. Network analysis can be used to identify the most central symptoms that are more likely to activate other symptoms and play crucial roles in the onset and maintenance of mental illnesses (15). A cross-sectional study in a psychiatric sample conducted before the pandemic showed the importance of sad mood and worry as the most central symptoms in the depression-anxiety network (16). In a recent network analysis study in Chinese clinicians, psychomotor symptoms, trouble relaxing, and worry were shown to be the central symptoms in the depression-anxiety network during the late stage of the COVID-19 pandemic (17). Network analysis also allows comparison between different networks, which would help us understand the changes in the network structure of depression and anxiety before and during the pandemic. By examining these changes in the network and identifying the central symptoms, we may be able to identify potential targets for clinical intervention during the COVID-19 pandemic (18), whose improvement may be accompanied by deactivation of the interactions with other psychopathology. Although several studies using network analysis have reported relationships between psychiatric symptoms at a time point before or after the COVID-19 outbreak, little is known about complex relationships among common psychiatric symptoms before and during the pandemic, and a comparison of the networks across the pandemic period in a clinical psychiatric sample is lacking.

The present study aimed to examine how the depression and anxiety symptom network changed during the COVID-19 pandemic in a naturalistic transdiagnostic sample of outpatients with non-psychotic mental illness. We used network analysis to estimate central symptoms and symptom-symptom interactions of depression and anxiety before the pandemic and during the first year of the pandemic, and compared their centrality and structure. Given the heightened fear and somatic concerns regarding COVID-19, we hypothesized that somatic symptoms would exhibit elevated centrality in the depression-anxiety network during the pandemic, while cognitive-affective symptoms would be the most central symptoms of the network before the pandemic.

## 2. Materials and methods

In this study, we included patients aged 18 years and above who first visited the psychiatric outpatient clinic of the Severance Hospital before and during the COVID-19 pandemic. For the “during pandemic group,” patients who first visited the clinic after a 3-month interval from the WHO declaration of worldwide pandemic in March were considered for participation. Therefore, we collected data of patients who first visited the clinic from June 1, 2020 to May 31, 2021 (1 year). For the “before pandemic group,” patients who first visited the clinic before the first case of COVID-19 was identified in Korea on January 20, 2020 were considered; we collected data of patients who first visited the clinic from January 20, 2019 to January 19, 2020, which is the same 1 year duration as the “during pandemic group.” All of the participants were assessed and diagnosed according to the Diagnostic and Statistical Manual of Mental Disorders, Fifth Edition (DSM-5). All participants were asked to answer standardized questions on social-demographic characteristics. Patients who were diagnosed with psychotic disorders or intellectual disabilities; had a history of brain injury, epilepsy, or other neurological diseases; or had other physical or psychiatric disabilities that hindered them from answering the questionnaires were excluded from the study. People with a history of COVID-19 were also excluded since they may have different psychological characteristics both indirectly *via* disruptive psychosocial changes and directly *via* neuropsychiatric sequelae after COVID-19 infection (3). The study protocol was approved by the Institutional Review Board (IRB) of the Severance Hospital, and all procedures of this study were conducted in accordance with the approved guidelines.

### 2.1. Assessment of depressive and anxiety symptoms

#### 2.1.1. Patient health questionnaire

To measure each patient’s depression, the PHQ-9 was administered. PHQ-9 contains nine items that correspond to the nine different symptoms from the diagnostic criteria of MDD from the DSM-IV: depressed mood/feeling down, loss of interest, sleep disturbance, change of appetite/weight, psychomotor agitation/retardation, loss of energy/fatigue, feelings of guilt/worthlessness, concentration difficulty/indecisiveness, and suicidal thoughts (19). Each of the nine items are scored from 0 (not at all) to 3 (nearly every day) depending on how often the subject had been disturbed by the symptom dimension during the preceding 2 weeks. Higher overall scores indicate greater depressive symptoms. The Korean version of the PHQ-9 has been shown to be a valid and reliable measure of depression (20), and the Cronbach’s alpha coefficient of PHQ-9 in our samples was 0.905.

#### 2.1.2. Beck anxiety inventory

To measure each’s patient’s anxiety, the BAI was administered. BAI contains 21 questions and each item is scored from 0 to 3 (i.e., not at all—severely), with higher scores indicating more severe anxiety symptoms (12). BAI comprises the somatic subscale which measures anxiety characterized by symptoms of physiological arousal, and the cognitive subscale which measures anxiety characterized by impaired cognitive functioning and fearful thoughts (21, 22). The somatic factor comprises the following items: numbness or tingling, feeling hot, wobbliness in the legs, dizzy or lightheaded, heart pounding or racing, unsteady, hands trembling, shaky, faint, face flushed, sweating, feeling of choking, difficulty breathing, and indigestion. The cognitive factor comprises the following items: unable to relax, fear of the worst happening, terrified, nervous, fear of losing control, scared, and fear of dying. The Korean version of the BAI has been shown to be a valid and reliable measure of anxiety (23). In our samples, Cronbach’s alpha coefficients of the somatic and cognitive subscales were 0.883 and 0.917, respectively, indicating good internal consistency.

### 2.2. Statistical analysis

Statistical analysis was completed using the Statistical Package for the Social Sciences version 26.0 and R. Sociodemographic and clinical characteristics of patients who first visited the clinic before and during the pandemic were compared using independent group *t*-test or Chi-square test (for categorial variables).

#### 2.2.1. Network estimation

The statistical software R was used to perform network analysis. Using the R-package qgraph (24), we estimated the network structure of depression and anxiety before and during the pandemic. In a network, variables are referred to as “nodes,” and “edges” are partial correlations between two nodes after controlling for all the other nodes in the network (24). The model was regularized by running the graphical least absolute shrinkage and selection operator, since a network with many parameters may lead to false-positive connections (25). Trivial, small, and partial correlations are driven to zero, revealing only relevant edges (26).

#### 2.2.2. Centrality

To examine and compare the importance of each node and edge in the network, centrality indices were calculated. The most commonly used centrality indices are strength, closeness, and betweenness. Strength centrality computes the sum of all edge weights to which a node is directly connected (27). Closeness centrality is the inverse of the weighted sum of distances between a particular node and other nodes in the network, and it measures the degree to which a node is indirectly connected to other nodes (27, 28). Betweenness centrality calculates the number of times that a node lies on the shortest path length between any two other nodes. The R-package bootnet was used to quantify the stability of centrality indices, which gives the correlation stability (CS) coefficients for each centrality index. It has been suggested that the CS-coefficient should not be below 0.25, and preferably above 0.5 (26). In this study, we only interpreted centrality indices with CS coefficients greater than 0.5.

#### 2.2.3. Network comparison analysis

The networks of depression and anxiety before and during the pandemic were compared using the R-package network comparison test (29). Specifically, network comparison test was used to test (1) whether the structure of the two networks was different and if the structure was different, which edges were different in strength, and (2) whether the overall level of connectivity was equal between the two networks. The current study used 1,000 permutations.

#### 2.2.4. Estimation of the required sample size

To provide an estimate of the required sample size, further analysis was performed using the *netSimulator* function of the R-package *bootnet*. The estimated correlation of edge weights, sensitivity, and specificity were computed between the original and estimated refitted network with various sample sizes before and during the COVID-19 pandemic. Details are presented in the Supplementary material.

## 3. Results

A total of 224 outpatients before the pandemic and 167 outpatients during the pandemic were included in the analysis. The demographic and clinical characteristics of the participants are presented in Table 1. The two groups did not show significant difference in age, sex distribution, years of education, PHQ-9 score, and BAI score. Among the primary categorical diagnosis of the participants, anxiety disorders were the most prevalent psychiatric disorders, followed by unipolar depressive disorders and somatic symptom disorders (Table 1). No difference in the proportion of primary diagnoses was found between the before and during pandemic groups. Of the participants, 52.5% had one or more psychiatric comorbidity.

**Table 1:** Clinical characteristics of participants.

	Before pandemic group (*n* = 224)	During pandemic group (*n* = 167)	*t* or χ^2^	*p*
Age (years)	44.23 ± 17.45	44.30 ± 16.70	–0.041	0.967
Male/female, n	77/147	44/123	2.885	0.089
Education (years)	13.92 ± 3.22	13.80 ± 3.17	0.352	0.725
PHQ-9	11.79 ± 7.64	12.42 ± 7.42	–0.819	0.413
BAI	22.17 ± 14.22	23.69 ± 14.99	–1.023	0.307
Primary diagnosis, n				
Anxiety	95 (42.41%)	73 (43.7%)	0.444	0.931
Depression	50 (22.3%)	34 (20.4%)		
Soma	44 (19.6%)	31 (18.6%)		
Others	35 (15.6%)	29 (17.4%)		

For age, education, PHQ-9, and BAI, mean ± standard deviation is presented. For the primary diagnostic group, the number and percentage of participants who were primarily diagnosed with anxiety disorders (Anxiety), unipolar depressive disorders (Depression), somatic symptom disorders (Soma), and other non-psychotic psychiatric disorders (Others) are presented. *t*-test for age, PHQ-9, and BAI. Chi-square test for sex and primary diagnosis. BAI, Beck Anxiety Inventory; PHQ-9, Patient Health Questionnaire-9.

### 3.1. Network before the COVID-19 pandemic

**Figure 1:**
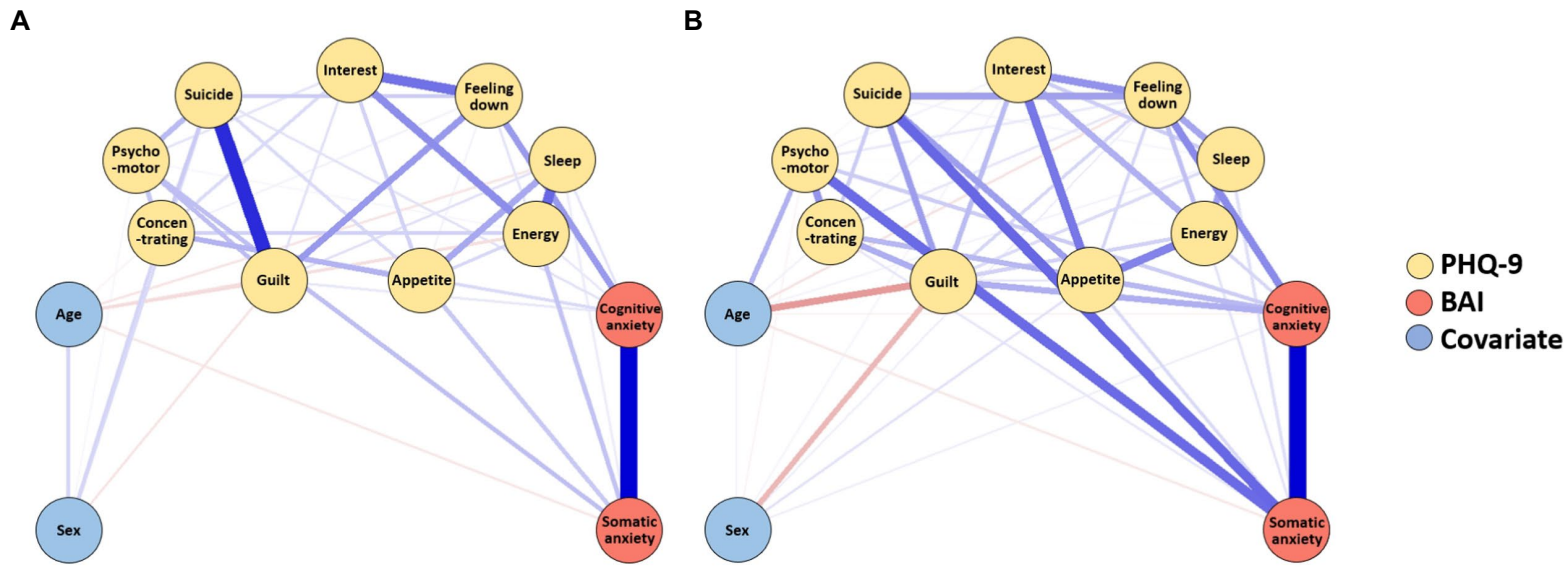
Estimated networks of depression and anxiety before **(A)** and during **(B)** the pandemic. Somatic anxiety, somatic subscale of the Beck Anxiety Inventory; Cognitive anxiety, cognitive subscale of the Beck Anxiety Inventory; Feeling down, item 1 of the Patient Health Questionnaire-9 (PHQ-9); Interest, item 2 of the PHQ-9; Sleep, item 3 of the PHQ-9; Appetite, item 4 of the PHQ-9; Psychomotor, item 5 of the PHQ-9; Energy, item 6 of the PHQ-9; Guilt, item 7 of the PHQ-9; Concentrating, item 8 of the PHQ-9, Suicide, item 9 of the PHQ-9. The blue edges indicate positive partial correlations, and the red edges indicate negative partial correlations. The thickness of the edges indicates the magnitude of the association.

**Figure 2:**
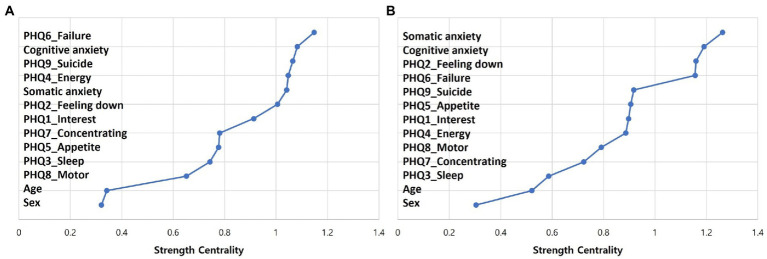
Standardized estimate of strength centrality before **(A)** and during **(B)** the pandemic. BAI_Som, somatic subscale of the Beck Anxiety Inventory; BAI_Cog, cognitive subscale of the Beck Anxiety Inventory; Feeling down, item 1 of the Patient Health Questionnaire-9 (PHQ-9); Interest, item 2 of the PHQ-9; Sleep, item 3 of the PHQ-9; Appetite, item 4 of the PHQ-9; Psychomotor, item 5 of the PHQ-9; Energy, item 6 of the PHQ-9; Guilt, item 7 of the PHQ-9; Concentrating, item 8 of the PHQ-9, Suicide, item 9 of the PHQ-9. The *X* axis shows strength centrality index of nodes, shown as standardized values z scores, and the *Y* axis shows nodes in order of strength centrality.

The estimated network of depression and anxiety before the pandemic is presented in Figure 1A. CS coefficients of the network were 0.674 for strength, 0.438 for closeness, and 0.129 for betweenness. As the CS coefficients of closeness and betweenness were inadequate, we interpreted strength as the primary index of centrality, which showed excellent stability. Strength centrality has been previously reported as a more stable and interpretable index than betweenness and closeness (26, 30). The standardized estimate of strength centrality of the network is presented in Figure 2A. Item 6 of the PHQ-9 (“Feeling bad about yourself or that you are a failure or have let yourself or your family down”) showed the highest strength centrality index, followed by item 9 of the PHQ-9 (“Thoughts that you would be better off dead, or of hurting yourself”), cognitive subscale of the BAI, and somatic subscale of the BAI.

### 3.2. Network during the COVID-19 pandemic

The estimated network of depression and anxiety during the pandemic is presented in Figure 1B. CS coefficients of the network were 0.593 for strength, 0.048 for closeness, and 0.126 for betweenness. As the CS coefficients of closeness and betweenness were inadequate, we interpreted strength as the primary index of centrality. The standardized estimate of strength centrality of the network is presented in Figure 2B. Somatic subscale of the BAI showed the highest strength centrality index, followed by cognitive subscale of the BAI, item 2 of the PHQ-9 (“Feeling down, depressed, or hopeless”), and item 6 of the PHQ-9 (“Feeling bad about yourself or that you are a failure or have let yourself or your family down”).

### 3.3. Network comparison analysis

**Figure 3:**
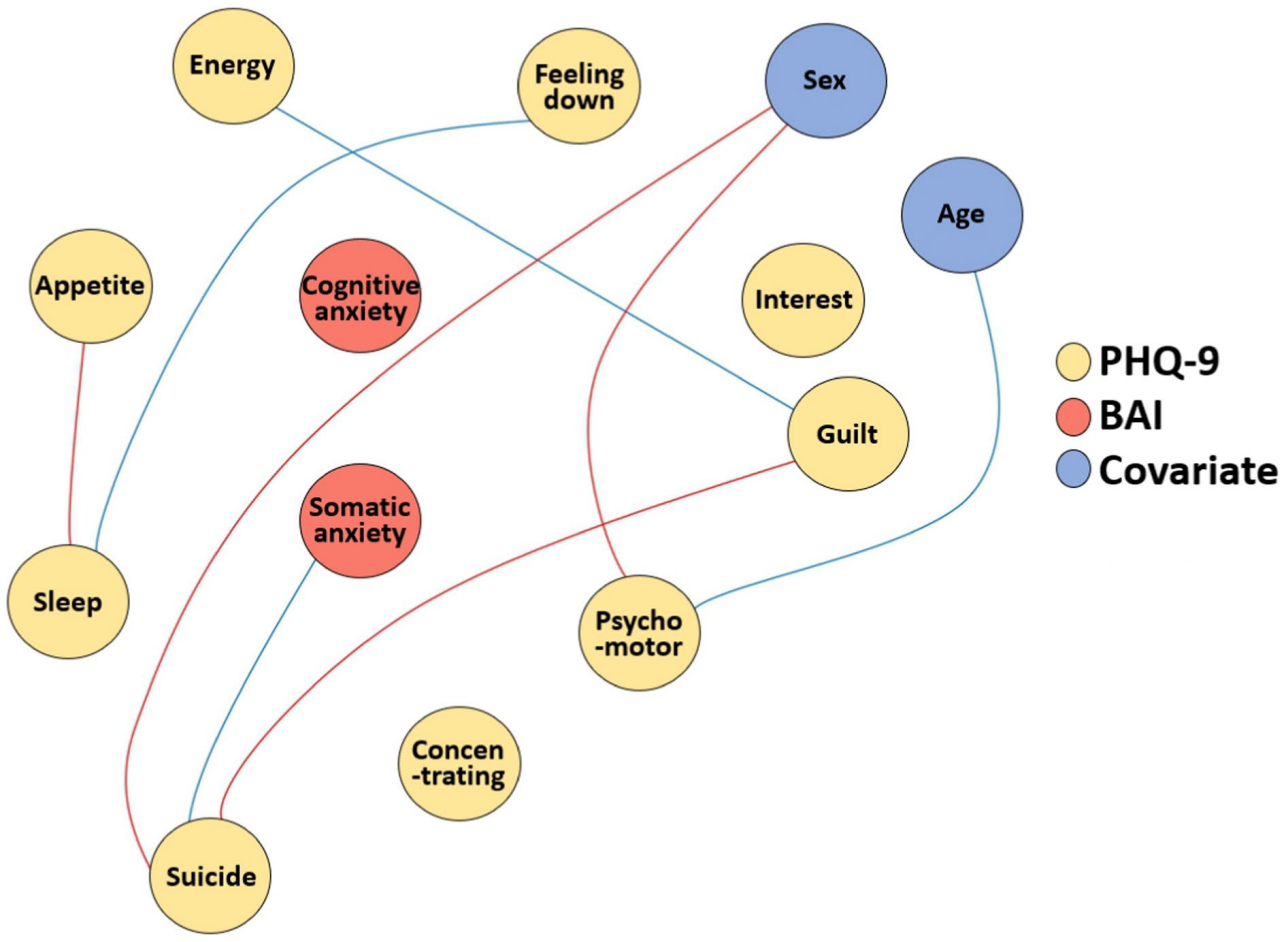
Network invariance test of the network of depression and anxiety before and during the pandemic BAI_Som, somatic subscale of the Beck Anxiety Inventory; BAI_Cog, cognitive subscale of the Beck Anxiety Inventory; Feeling down, item 1 of the Patient Health Questionnaire-9 (PHQ-9); Interest, item 2 of the PHQ-9; Sleep, item 3 of the PHQ-9; Appetite, item 4 of the PHQ-9; Psychomotor, item 5 of the PHQ-9; Energy, item 6 of the PHQ-9; Guilt, item 7 of the PHQ-9; Concentrating, item 8 of the PHQ-9, Suicide, item 9 of the PHQ-9. The blue edges indicate significantly increased partial correlations, and the red edges indicate significantly decreased partial correlations.

The two networks were compared regarding network properties. Network invariance test showed that there was a statistically significant difference in the global structure of the two networks (*p* = 0.016), while the global strength invariance test showed that there was no difference in the global strength of the two networks (*p* = 0.913). Therefore, each edge of the two networks were compared to see if there were any local differences. The edges that showed statistically significant differences before and during the pandemic are displayed in Figure 3. Notably, somatic subscale of the BAI, which showed the highest strength centrality index during the pandemic, showed significantly increased correlation with item 9 of the PHQ-9 (“Feeling bad about yourself or that you are a failure or have let yourself or your family down”; *p* = 0.002).

## 4. Discussion

In this study, we compared the networks of depression and anxiety before and during the COVID-19 pandemic in Korea. We observed a structural change between the networks before and during the pandemic and identified somatic anxiety as a central symptom in the network of depression and anxiety in the context of the pandemic. To our knowledge, this is the first study to directly compare the psychological networks before and during the pandemic in a sample of psychiatric outpatients.

Notably, while feeling of guilt was the most central symptom in the network before the pandemic, somatic anxiety emerged as the most central symptom in the network during the pandemic. Somatic anxiety, which is the physical manifestation of anxiety, may include symptoms such as gastrointestinal discomfort, chest pain, fatigue, dizziness, and headache (31). The importance of somatic anxiety among symptoms of depression and anxiety during the pandemic, as shown in our study, is in line with the results of previous studies. One study utilized network analysis to compare the network of depression and anxiety of the general population, during and after the peak of COVID-19 in China, and showed that during the outbreak, psychomotor symptoms such as impaired motor skills, restlessness, and inability to relax, as measured by the Generalized Anxiety Disorder Assessment (GAD-7), exhibited high centrality in the network (32). Similarly, in a study on mental health of health care workers during the COVID-19 pandemic, workers working in COVID-19 prevalent areas had significantly higher somatic and anxiety symptoms compared to those of workers at non-prevalent areas (33). In another study of the United Kingdom general population, COVID-19 related anxiety was strongly associated with somatic symptoms such as fatigue and gastrointestinal discomfort (34).

Emergence of somatic anxiety as the central symptom over the course of the pandemic may be explained by several possible mechanisms. First, it is possible that during pandemics, enhanced health anxiety, which is characterized by obsessive worries of having a serious condition and misinterpretations of benign bodily sensations or physiological arousal as a serious medical illness (35), may contribute to increased somatic anxiety. With the *rapid spread* of *COVID*-*19*, people were typically exposed to a great amount of health-related media information and commonly *worried* about their *health* (1). Although being worried about contracting an illness may be helpful for mitigating infection risk during the pandemic, obsessive checking of bodily signs of illness could enhance awareness of bodily sensations and eventually lead to heightened experiences of somatic anxiety, in forms of palpitations, gastrointestinal discomfort, and muscle tension. In addition, as people with high health anxiety tend to have *catastrophic interpretation about bodily* sensations and overestimation bias of the likelihood of illness and death (36), they could experience high physiological arousals. It should be noted that in our previous study, health worry appeared to be an important bridge symptom that connected COVID-19 anxiety and other clinical psychopathology (37). Second, enhanced attentional bias to negative information during the pandemic may contribute to enhanced somatic anxiety. Attentional bias, which refers to prioritization of certain types of stimuli to increase our ability to process this information (38), has been suggested to play a key role in the development and maintenance of anxiety (39). Social isolation and loneliness, a major concern during the COVID-19 quarantine period, may exaggerate attentional bias to negative emotion and hypervigilance to threats (40, 41). In one study conducted during the COVID-19 pandemic, negative attention bias was found to be significantly correlated with physical anxiety sensitivity (42). Furthermore, health anxiety and attentional bias toward heath threat-related information have been reported to be closely linked (43). A recent study during the COVID-19 pandemic also showed that high health anxiety was associated with high attentional bias towards virus-related stimuli in a nonclinical population (44). Further research is required to confirm the present findings of somatic anxiety and to gain a better understanding of the relationships among health anxiety, attentional bias, and clinical psychopathologies.

In addition, in the network comparison analysis, there was a statistically significant increase in edge strength between somatic anxiety and suicidal ideation, concomitant with a significant decrease in edge strength between inappropriate guilt and suicidal ideation. This indicates that relative association of somatic anxiety on suicidal ideation increased during the pandemic. Somatic anxiety has been shown to be associated with suicidal ideation in previous studies (45, 46), but to the best of our knowledge, this is the first study to report a strong association for somatic anxiety with suicidal ideation in the context of the COVID-19 pandemic. It is possible that during the time of pandemic, somatic manifestation of anxiety may be misinterpreted and attributed to COVID-19 (1), which in turn may increase anxiety and contribute to elevated suicidal ideation. Thus, cognitive behavioral approach to somatic anxiety that involves breathing training and muscle relaxation, such as in cognitive behavioral therapy for panic disorder, may be helpful for patients in dealing with somatic anxiety and may lead to reductions in suicidal ideation in such patients.

Interestingly, the network comparison analysis also showed that there was a statistically significant increase in edge strength between feeling down and sleep, concomitant with a significant decrease in edge strength between appetite and sleep. The reduced tie between symptoms of appetite and sleep over the course of the COVID-19 pandemic may indicate that sleep disturbance during the pandemic could be related to stress-related symptoms in response to environmental factors, such as social disconnection, disruption of daily routine, and financial problems in the context of COVID-19, rather than a neurovegetative feature of depression. It may be explained by the concept of reactive versus endogenous depression (47); the during pandemic group in this study may have relatively more subjects with reactive depression rather than endogenous depression, indicating co-occurrence of sleep disturbances and appetite symptoms (48), whereas the before pandemic group may have relatively less subjects with reactive depression.

There are some study limitations that should be noted. First, while our study showed adequate stability for strength centrality of the network, the present sample size was insufficient to obtain stable coefficients for closeness and betweenness centralities. Second, the results from a clinical psychiatric sample in a Korean tertiary hospital may not generalize to the entire Korean population and other populations with different COVID-19 situation and cultural differences in responses to COVID-19. Third, although our findings of direct comparison between networks before and during the pandemic bring to light the COVID-19 pandemic’s impact on depression and anxiety symptoms, the two cross-sectional network analyses of individuals at one point in time cannot demonstrate causal relationships among measured variables and cannot be assumed to generalize to the intraindividual level. Finally, we have unmeasured potential confounders including physical health conditions, health behaviors, and physical activities, which may have an influence on depression and anxiety network. Future research in a larger sample is needed by incorporating a broader set of risk factors and confounders.

In conclusion, the present study examined the COVID-19 pandemic’s impact on networks of depression and anxiety symptoms in a naturalistic transdiagnostic sample of psychiatric outpatients with non-psychotic mental illness. The networks of depression and anxiety symptoms were shown to be different when compared before and during the pandemic. Unlike the network before the pandemic, somatic anxiety emerged as a robust central symptom of the network during the pandemic. The findings indicate that the COVID-19 pandemic has brought a significant change in the depression and anxiety symptom network, and somatic anxiety may serve as a potential effective target for psychiatric intervention in the context of the pandemic.

## Data availability statement

The datasets presented in this article are not readily available because the data is personal medical information. Requests to access the datasets should be directed to corresponding author.

## Ethics statement

The studies involving human participants were reviewed and approved by Institutional Review Board of the Severance Hospital. Written informed consent for participation was not required for this study in accordance with the national legislation and the institutional requirements.

## Author contributions

STK performed the analysis, interpreted data, and wrote the original draft. JHS and CIP interpreted data and revised the manuscript. SJK analyzed the data and revised the manuscript. JIK conceived and designed the study, performed the analysis, and wrote the manuscript. All authors contributed to the article and approved the submitted version.

## Funding

This work was supported by a National Research Foundation of Korea (NRF) grant funded by the Korean government (NRF-2019R1A2C1084611).

## Conflict of interest

The authors declare that the research was conducted in the absence of any commercial or financial relationships that could be construed as a potential conflict of interest.

## Publisher’s note

All claims expressed in this article are solely those of the authors and do not necessarily represent those of their affiliated organizations, or those of the publisher, the editors and the reviewers. Any product that may be evaluated in this article, or claim that may be made by its manufacturer, is not guaranteed or endorsed by the publisher.
