# Supplementary material for: COVID-19 pandemic’s impact on networks of depression and anxiety in naturalistic transdiagnostic sample of outpatients with non-psychotic mental illness
Source: Front Psychiatry. 2023 Mar 13;14:1118942. doi: 10.3389/fpsyt.2023.1118942 (PMC10040589; doi:10.3389/fpsyt.2023.1118942)
Supplement: Supplementary file 1 [file Data_Sheet_1.docx]

**Supplementary Information**


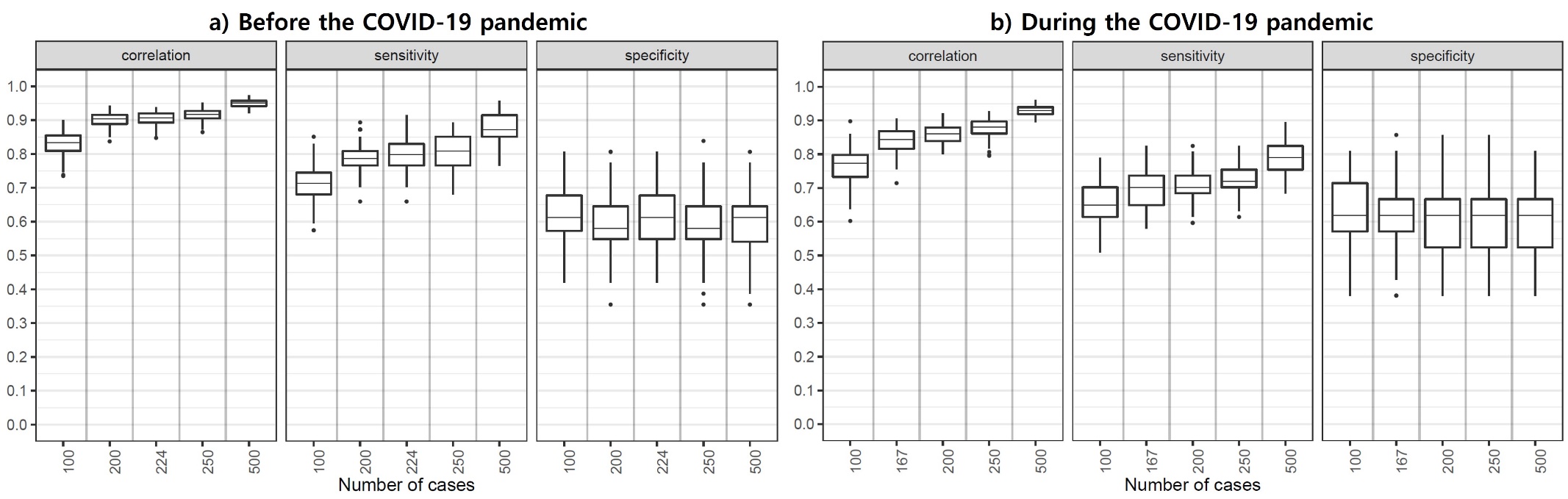


**Supplementary Figure S1.** Estimation of the required sample size. Boxplots represent the estimated correlation of edge weights, sensitivity, and specificity between the original and estimated refitted network with various sample size a) before and b) during the COVID-19 pandemic. For the original 13-node network, a sample size of 224 for the before pandemic network would achieve a correlation between “true” and estimated networks around 0.9 for edge weights and strength. A sample size of 167 for the during pandemic network would achieve a correlation between “true” and estimated networks above 0.8 for edge weights and strength.
